# Implications of the c.1201C > G (p.Arg401Gly) mutation in *FGG* gene on fibrinogen stability and function

**DOI:** 10.3389/fmed.2025.1668007

**Published:** 2025-09-15

**Authors:** Jingyi Lu, Zeyi Xiang, Yonglong Ye, Wei Chen, Jieyi Tan, Bin Kuang, Jun Liu

**Affiliations:** ^1^Department of Laboratory Medicine, Dongguan Hospital of Guangzhou University of Chinese Medicine, Dongguan, China; ^2^Department of Nephrology, Dongguan Hospital of Guangzhou University of Chinese Medicine, Dongguan, China

**Keywords:** congenital dysfibrinogenemia, *FGG*, c.1201C > G mutation, fibrinogen, sanger sequencing

## Abstract

Congenital dysfibrinogenemia, a rare coagulation disorder characterized by decreased fibrinogen activity while antigen level is usually normal. We conducted a study on a three-generation family comprising 15 members, among whom three individuals were diagnosed with this condition. This study aimed to elucidate the genetic and structural basis of dysfibrinogenemia in this family. Coagulation assays revealed significantly reduced fibrinogen levels in the proband, his father, and his son, with mild prolongation of PT and TT. Despite normal liver and kidney function, recurrent nosebleeds were reported in the proband and his son. Whole-exome sequencing identified a novel variant (c.1201C > G, p.Arg401Gly) in the *FGG* gene, confirmed by Sanger sequencing. Structural analysis indicated that the mutation disrupted hydrogen bonding in the FGG protein, compromising its stability and potentially impairing fibrinogen assembly. Scanning electron microscopy of fibrin clots from affected individuals demonstrated a reduced fiber network density compared to healthy controls, further supporting the mutation’s impact on fibrinogen structure. These findings suggest that the p.Arg401Gly mutation in the *FGG* gene is a likely contributor to the observed dysfibrinogenemia, affecting both protein stability and fibrin network integrity. This study is the first to document the c.1201C > G mutation in the *FGG* gene, resulting in the substitution of arginine with glycine at the 401st position, consequently impairing fibrinogen function. This discovery holds significant implications for genetic counseling and prenatal genetic diagnosis.

## Introduction

Congenital dysfibrinogenemia (CD) is a disorder caused by defects in the fibrinogen gene, primarily resulting in structural abnormalities and impaired function of fibrinogen (1). The disease follows an autosomal dominant inheritance pattern, with the majority of patients exhibiting heterozygous missense mutations (2). The clinical presentation of CD is highly variable: approximately 50% of patients are asymptomatic and are identified incidentally during routine physical examinations; around 25% exhibit a bleeding tendency; and thrombotic events occur in about 20% of patients (3). Notably, pregnant women with CD may experience complications such as spontaneous abortion or placental abruption. An analysis of 140,000 genomes estimated the global prevalence of autosomal dominant fibrinogen defects to be approximately 11 per 1,000, with no significant racial differences (4). Patients with hereditary fibrinogen defects are typically identified during routine examinations or preoperative laboratory screenings, primarily characterized by decreased fibrinogen activity (Clauss method) (1, 5, 6).

A diagnosis of CD should be considered when the ratio of fibrinogen activity to antigen is less than 0.7, provided that the antigen level is normal. Additionally, the fibrinogen ratio measured by prothrombin time (PT) derivatization compared to the Clauss method serves as an important tool for screening dysfibrinogenemia (6). Studies indicate that when this ratio exceeds 1.43, the diagnosis of hereditary dysfibrinogenemia exhibits 100% sensitivity and specificity (7). Fibrinogen is a hexamer composed of three polypeptide chains: Aα, Bβ, and *γ* (8). Its derivative, fibrin, plays a critical role in various biological processes, including cell-matrix interactions, angiogenesis, blood coagulation, wound healing, inflammatory responses, and tumorigenesis. Mutations in the genes encoding these chains—*FGA*, *FGB*, and *FGG*—responsible for CD have been strongly associated with the development of dysfibrinogenemia; however, the precise mechanisms of pathogenesis remain largely unclear. Previous case studies suggest that variants in the *FGG* gene may lead to decreased fibrinogen levels by destabilizing fibrin and impairing its assembly (9). Therefore, thorough analysis of specific families is essential to elucidate how *FGG* gene variants affect disease occurrence and progression, as well as to enhance understanding of its genetic and biological underpinnings.

In this study, we collected samples from 15 family members spanning three generations, including three patients with CD. Routine testing showed that the fibrinogen levels (Clauss method) in these patients were significantly lower than the normal reference range, accompanied by markedly elevated IgE levels. Whole-exome sequencing subsequently identified a c.1201C > G mutation in the *FGG* gene, resulting in the substitution of arginine (Arg) with glycine (Gly) at position 401. Structural prediction and electron microscopy analyses indicated that this mutation led to significant structural alterations in fibrinogen. Collectively, our study enhances the understanding of hereditary abnormal fibrinogenemia linked to mutations in the *FGG* gene.

## Materials and methods

### Specimen collection and processing

Venous blood samples were obtained from family members of the patients following the collection manual and immediately centrifuged at 1,500 g for 15 min.

### Coagulation assays and thromboelastography

Coagulation assays were conducted using the Stago STA R Max system (France) along with traceable reagents to determine PT (REF00667, DIAGNOSTICA STAGO S. A. S., France), thromboplastin time (TT) (REF00669, DIAGNOSTICA STAGO S. A. S., France), activated partial thromboplastin time (APTT) (REF00595, DIAGNOSTICA STAGO S. A. S., France), and fibrinogen levels (REF00674, DIAGNOSTICA STAGO S. A. S., France). Additionally, Sysmex CS5100 (Japan) was utilized for PT (Thromborel S, Siemens Healthcare Diagnostics Products GmbH, Germany) and fibrinogen-C assays, using traceable reagents for PT derivatization and fibrinogen-C. Thromboelastography measurements were conducted with a T-400 Thromboelastography Hemostasis Analyzer (Yangpu, China).

### Genetic analysis

Genetic assays were performed using the Illumina sequencing platform, covering the exons of known genes in the human genome along with 5 bp of upstream and downstream sequences. Genomic DNA was processed utilizing the xGEN Exome Research Panel v1.0 capture kit (Integrated DNA Technologies, United States) in conjunction with the TruePrep Flexible DNA Library Prep Kit for Illumina (Vazyme, China). This process achieved an average sequencing depth of 90X, with 98% of target sequences exceeding a depth of 20X, ensuring high sensitivity and accuracy. NG_008834.1 and NM_000509.6 were used as the reference sequences for this study. BWA was employed to align the sequenced fragments to the UCSC hg19 reference genome, and the GATK software suite was used to analyze the data and identify genetic variants. Functional annotation of the variants was conducted using VEP software, and their clinical significance was assessed in conjunction with authoritative databases such as ClinVar, OMIM, HGMD, and gnomAD.

### Variant structure prediction

The amino acid sequence information for FGG proteins was retrieved from the UniProt database.^1^ The mutant sequences were then modeled using both the Swiss Model^2^ and AlphaFold2 to predict structural changes resulting from the identified mutations.

### Scanning Electron microscopy observation

In this study, 50 microliters (50 μL) of plasma samples were collected from proband, the patient’s son, and healthy controls. To induce fibrin clot formation, thrombin was added to each sample to achieve a final concentration of 2 U/mL. The mixtures were incubated at a constant temperature of 37°C for 3 h to allow for sufficient fibrin clot formation. After incubation, the fibrin clots were carefully isolated and their ultrastructure was examined using a scanning electron microscope (8100, Hitachi, Japan).

## Results

### Patient data analysis

**Figure 1:**
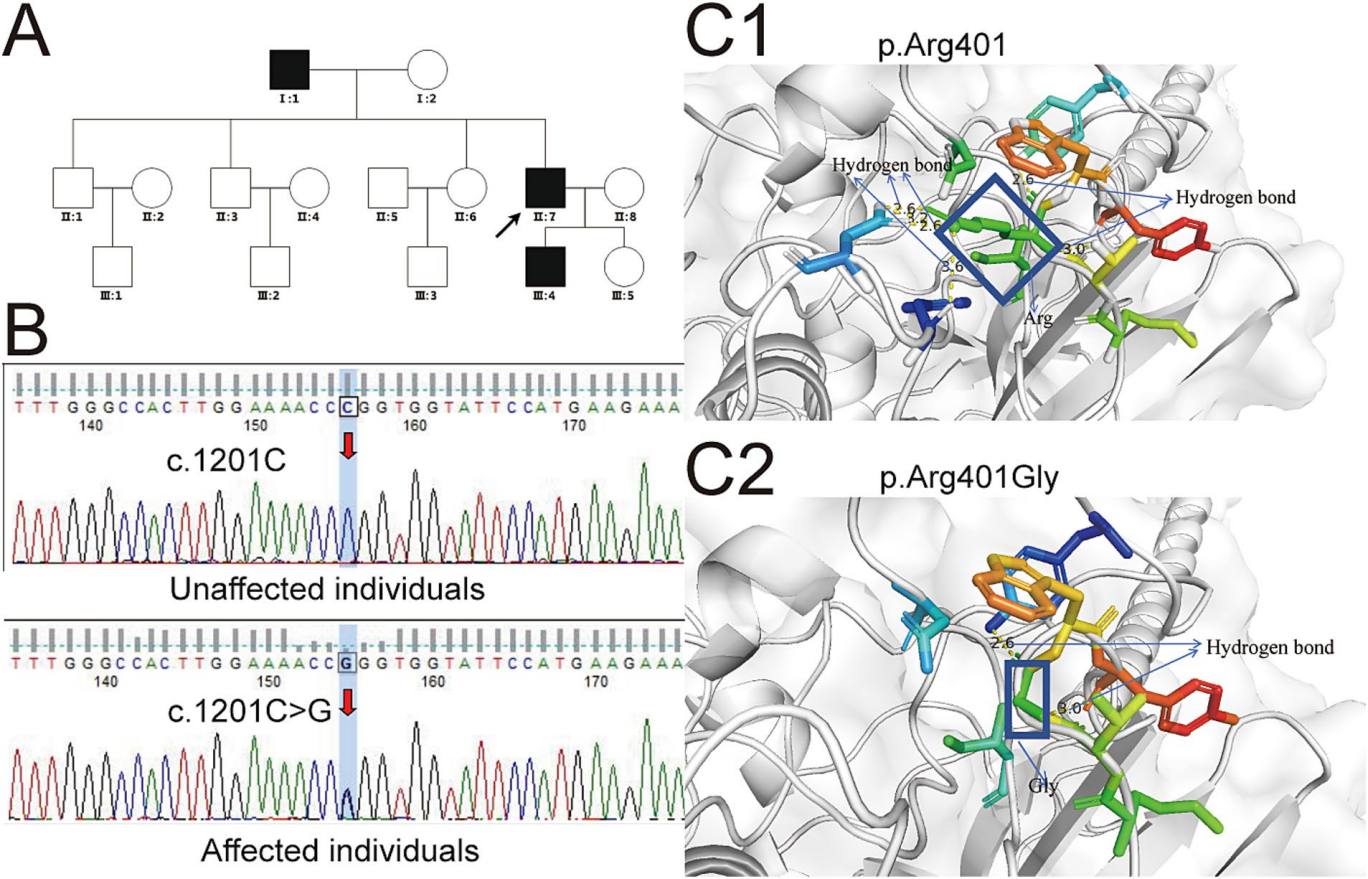
Genetic variations in the *FGG* gene associated with familial dysfibrinogenemia and implications for protein structure prediction. **(A)** The family pedigree illustrating hereditary low fibrinogen levels is depicted, with solid circles indicating affected individuals and hollow circles representing unaffected individuals. The arrow indicates the proband. **(B)** Sanger sequencing identified the specific locus of the mutation within the *FGG* gene. **(C)** The structural prediction model of the FGG protein was developed utilizing the Swiss-Model platform. Yellow dotted lines denote hydrogen bonds, with numerical annotations indicating bond lengths. Additional colored bars represent surrounding amino acid residues, while the coiled-coil domain of fibrinogen is illustrated using a wheat color scheme. **(C1)** The structure of the normal p.Arg401 in the *γ* chain of fibrinogen, as compared with the structure of the variant p.Arg401Gly, which is shown in **(C2)**. In **(C1)**, the rectangle denotes the structure of Arg, whereas in **(C2)**, the rectangle represents the structure of Gly. The c.1201C > T mutation results in a substitution of arginine (a positively charged amino acid) with tryptophan (a large, hydrophobic amino acid). This significant change in chemical properties likely disrupts the overall structure and stability of the fibrinogen γ chain, leading to reduced levels of functional fibrinogen. In contrast, the c.1201C > G mutation results in a substitution of arginine with glycine (a small, neutral amino acid). This change might not significantly affect the overall stability or synthesis of the γ chain but could alter its conformation in a way that impairs its functional interactions.

This study involved a three-generation family consisting of 15 members, three of whom were diagnosed with dysfibrinogenemia (Figure 1A). The proband, a 44-year-old man, was identified with an abnormality during routine testing of fibrinogen levels. Following this discovery, his parents, two brothers, one sister, son, and daughter underwent coagulation assessments. The results indicated that both his father and son also exhibited reduced fibrinogen levels. The proband reported no history of bleeding or symptoms, such as mucosal bleeding or easy bruising; however, both he and his son experienced recurrent nosebleeds that were difficult to control. Liver and kidney function tests performed on the index patient and other affected family members yielded normal results, suggesting that the underlying process of fibrinogen production was not defective. In the coagulation tests, both the proband and other affected family members demonstrated mild prolongation of PT and TT. Specifically, the fibrinogen levels recorded were 0.91 g/L for the proband, 0.85 g/L for his son, and 0.98 g/L for his father, all significantly below the normal range (2.0–4.0 g/L). In contrast, the fibrinogen levels derived from the PT algorithm were higher, measuring 1.82, 1.84, and 1.8 g/L, respectively. Additionally, to achieve a more precise evaluation of fibrinogen levels, we employed an automated biochemical analyzer (BS-350E, Mindray) for the assessment of fibrinogen antigen concentrations. The results indicated that the fibrinogen antigen levels in the three family members were 3.03, 3.11, and 2.96 g/L, respectively (Table 1).

**Table 1:** Results of coagulation, thromboelastography, and liver function tests for the proband, his father, and his son.

Characteristics	Test	I:1 (proband’s father)	II:7 (proband)	III:4 (proband’s son)	Reference interval
Coagulation testing results	PT	14.7	14.5	14.8	11.0–14.5 (second)
	APTT	34	33.9	35.1	28.0–43.0 (second)
	TT	24.0	23.6	22.0	14.0–21.0 (second)
	Fibrinogen	0.95	0.91	0.85	2.0–4.0 (g/L)
	Fibrinogen: PT-derived	1.82	1.84	1.8	2.0–4.0 (g/L)
	Fibrinogen: Ag	3.03	3.11	2.96	2.0–4.0 (g/L)
Thromboelastography	R		4.4	3.6	5.0–10.0 (minute)
	K		2.9	2.4	1.0–3.0 (minute)
	MA		45.2	44.7	50.0–70.0 (millimeter)
Liver function	Prealbumin	289	312	186	180.0–350.0 (mg/L)
	Total protein	77.5	72.3	67.7	65.00–85.00 (g/L)
	Albumin	45.2	43.6	44.2	40.00–55.00 (g/L)
	Total bilirubin	17.5	18.3	10.5	0.00–21.00 (μmol/L)

PT, Prothrombin Time; APTT, Activated Partial-Thromboplastin-Time; TT, Thrombin Time; R, Rection Time; K, K value; MA, Maximal amplitude.

### Genetic analysis

To investigate the underlying cause of dysfibrinogenemia in the family, whole-exome sequencing was conducted. This analysis identified a novel variant locus (c.1201C > G, p.Arg401Gly) in the *FGG* gene present in the proband, his father, and his son. A secondary validation of this variant was performed using Sanger sequencing, which yielded results highly consistent with those of the whole-exome sequencing (Figure 1B). To elucidate the impact of this variant on the structure and function of the FGG protein, we analyzed the predicted structural domains of both wild-type and mutant FGG proteins. In the wild-type protein, the Arg at position 401 is located near a neighboring *α*-helix region and forms hydrogen bonds with it. However, after the mutation to Gly, the shorter side chain of glycine fails to maintain the original hydrogen bonding structure, leading to the disruption of these bonds (Figure 1C). Specifically, wild-type arginine 401 can form 6 hydrogen bonds with 4 neighboring residues, while the mutant can only form 2 hydrogen bonds with 2 residues. This significant difference in hydrogen bond number and distribution critically affects the stability of the FGG protein. As a crucial component of fibrinogen, the stability of FGG is essential for the normal assembly and function of fibrinogen. Thus, it is hypothesized that the p.Arg401Gly mutation may disrupt fibrinogen assembly by compromising FGG protein stability, leading to decreased fibrinogen levels. In addition, adhering to the ACMG/AMP guidelines for variant interpretation, we performed a comprehensive classification of the variants identified in this study. Drawing on the evidence gathered, we have classified the c.1201C > G (p.Arg401Gly) variant in the *FGG* gene as likely pathogenic (Table 2).

**Table 2:** Overview of c.1201C > G (p.Arg401Gly) in *FGG* for ACMG/AMP criteria and evidence codes.

Evidence code	Simplified criterion description	Extension and other comments
PM1	The variant is located in a known pathogenic hot spot or critical functional domain.	The 401st arginine of the *FGG* gene is highly conserved, and variants at this site are known to be associated with congenital dysfibrinogenemia.
PM2	The variant is absent from public databases (e.g., gnomAD) or is extremely rare.	The variant was not found in the gnomAD database, indicating that it is extremely rare in the general population.
PP3	Multiple computational tools predict that the variant is likely to have a deleterious effect on protein function.	Both Swiss Model and AlphaFold2 predict that the variant is likely to impair protein function.
PP4	The patient’s phenotype is highly consistent with the disease known to be associated with pathogenic variants in the *FGG* gene.	The patient presented with recurrent nosebleeds, and coagulation function tests showed reduced fibrinogen activity, which is consistent with the clinical manifestations of congenital dysfibrinogenemia.
PP5	Functional studies support the pathogenicity of the variant.	Structural analysis shows that the variant disrupts hydrogen bonding in the FGG protein, affecting protein stability. Scanning electron microscopy revealed reduced fiber network density in the patient’s fibrin clots, further supporting the impact of the variant on fibrinogen structure and function.

### Ultrastructural examination

**Figure 2:**
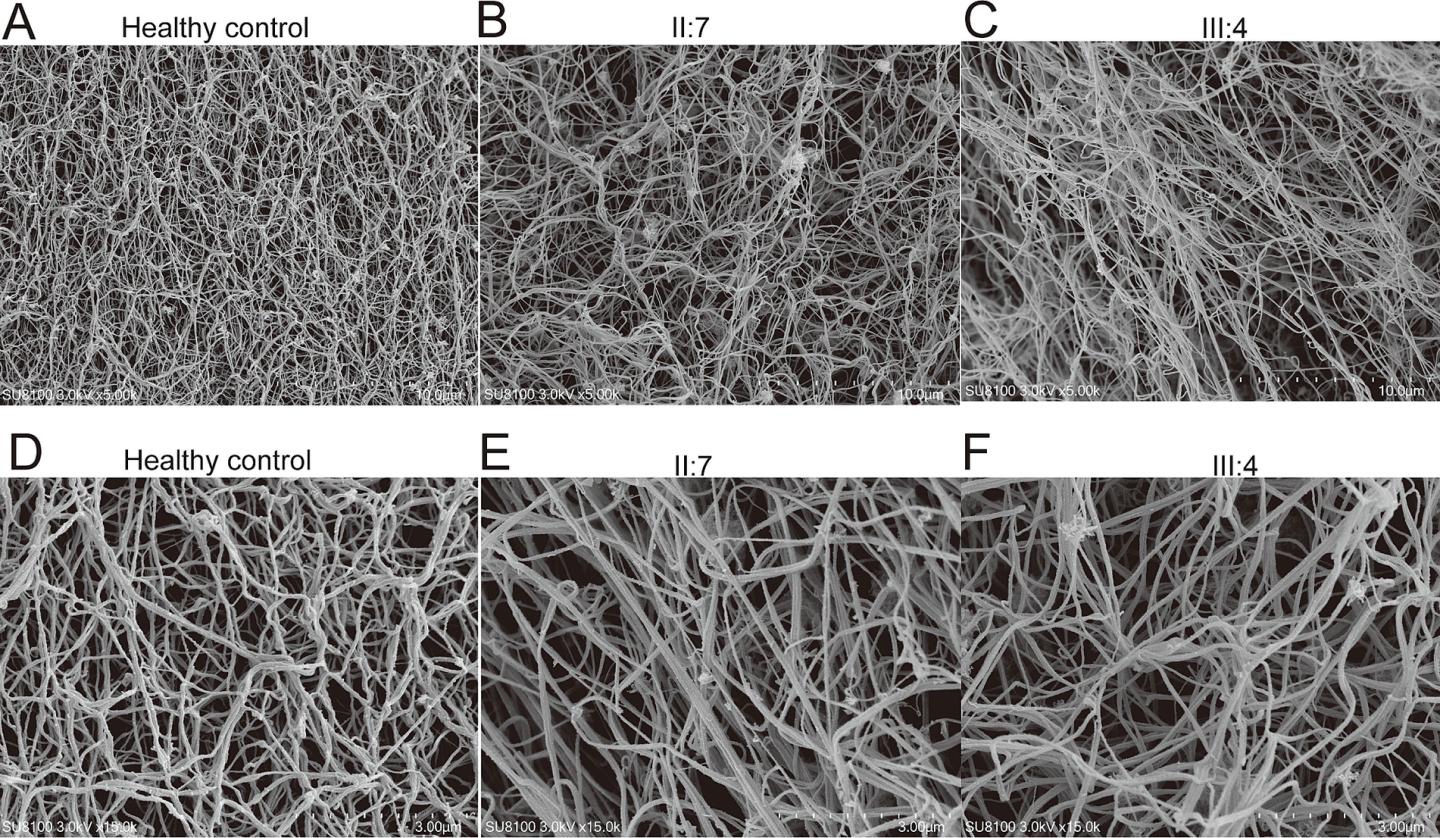
Examination of fibrin clots using scanning electron microscopy. 5,000-fold electron microscope examination of fibrin clots from healthy control **(A)**, patient II:7 **(B)**, and patient III:4 **(C)**. 15,000-fold electron microscope examination of fibrin clots from healthy control **(D)**, patient II:7 **(E)**, and patient III:4 **(F)**.

To verify the mutation’s effect on fibrin structure at the microstructural level, samples were collected from healthy individuals (fibrinogen: 3.11 g/L) as well as from two patients carrying the mutation, and fibrin clots were prepared. Scanning electron microscopy was subsequently employed to examine their internal structures in detail. The observations revealed that the density of the fiber network was significantly lower in patients II:7 and III:4 compared to healthy controls. This finding suggests that the mutation has negatively impacted the structural integrity of fibrinogen, contributing to the observed dysfibrinogenemia (Figure 2).

## Discussion

CD is a hereditary disorder primarily characterized by dysfunction in blood coagulation (10). Patients often experience recurrent bleeding and difficulty achieving hemostasis (11). A study involving 102 patients with CD indicated that 68.6% of these individuals were asymptomatic, 27.5% exhibited bleeding symptoms, and only 3.9% developed thrombosis (12). The pathogenesis of CD is closely associated with mutations in the *FGA*, *FGB*, and *FGG* genes, leading to abnormal fibrinogen function. To date, more than 450 mutations in the fibrinogen genes have been documented globally,^3^ with 212 of these mutations resulting in single amino acid changes. Among these mutations, those in the *FGA* gene are the most common, followed by mutations in the *FGG* gene (13). The primary etiology of CD is attributed to heterozygous missense mutations in the *FGA* and *FGG* genes, which are responsible for 99.3% of cases (14). The specific location of mutations within the fibrinogen genes significantly influences the clinical manifestations of CD. For instance, the p.Arg554Cys mutations in the *FGA* gene, the p.Ala68Thr mutation in the *FGB* gene, and the p.Asp364Val mutation in the *FGG* gene have all been linked to thrombosis (15). It is also important to note that even with identical mutations, clinical manifestations can vary among individuals due to personal biological differences. This variability underscores the complexity of clinical outcomes in patients with CD.

In the present study, we conducted an investigation of a three-generation family affected by CD and identified three patients with a marked decrease in fibrinogen levels. Specifically, their fibrinogen levels were recorded at 0.98, 0.91, and 0.85 g/L, all significantly below the normal reference range. Additionally, fibrin levels calculated using the PT derivatization algorithm were also lower than normal, measuring 1.9, 1.84, and 1.8 g/L. Previous research indicates that individuals with fibrinogen levels above 1.0 g/L typically do not exhibit significant clinical symptoms (16). However, a critical threshold exists; when fibrinogen levels dip below 0.5 g/L, patients may be prone to spontaneous or traumatic bleeding (17). While the average fibrinogen levels of these three patients were low, they had not yet fallen below this critical threshold, resulting in an absence of typical spontaneous bleeding symptoms. Despite this, all three patients demonstrated mild prolongation of TT and PT, indicating some degree of coagulation dysfunction, although it was not severe enough to cause spontaneous bleeding. Notably, all three patients reported multiple episodes of nosebleeds, which were difficult to control. This observation suggests that their coagulation dysfunction may yield a more pronounced bleeding tendency under specific circumstances, such as minor irritations of the fragile nasal mucosa, even though their overall fibrinogen levels had not reached the critical threshold for severe spontaneous bleeding. This finding emphasizes that when evaluating the bleeding risk in patients with CD, it is crucial to consider not only the absolute fibrinogen levels but also the patient’s clinical presentation and context-specific factors. A comprehensive assessment of the patient’s condition and potential bleeding risk should incorporate both laboratory values and individual symptoms to ensure a complete understanding of their clinical status. Furthermore, it has been observed in previous studies that the c.1201C > T mutation in the *FGG* gene results in an amino acid substitution from p.Arg401 to p.Trp401 (18). This mutation is associated with hereditary hypofibrinogenemia with liver storage, which is characterized by reduced fibrinogen levels and abnormal accumulation of fibrinogen within hepatic cells. In the present study, the c.1201C > G mutation predominantly results in congenital dysfibrinogenemia, with the patient’s fibrinogen antigen levels remaining within the normal range. This discrepancy may be attributed to the fact that the c.1201C > T mutation replaces arginine, a positively charged amino acid, with tryptophan, a large and hydrophobic amino acid. Such a substantial alteration in chemical properties may disrupt the overall structure and stability of the fibrinogen *γ* chain, thereby leading to reduced functional fibrinogen levels. Conversely, the c.1201C > G mutation substitutes arginine with glycine, a small and neutral amino acid. This substitution may not significantly impact the overall stability or synthesis of the γ chain; however, it may induce conformational changes that affect its functional interactions.

In the present study, we conducted thromboelastography on affected patients and their sons, revealing a significant reduction in both the Angle and Maximum Amplitude (MA) values. This finding indicates diminished fibrinogen activity and compromised fibrin function (19). The stability of a clot is largely dependent on the collaborative actions of platelets and fibrinogen. Specifically, platelets enhance clot stability by binding to fibrin through glycoprotein IIb/IIIa on their surfaces and promoting clot contraction through cytoplasmic motor proteins within the platelets (20). In the family we studied, the MA values were below the normal reference range despite normal platelet counts and function. This discrepancy suggests that the abnormal fibrinogen present is likely unable to bind effectively to activated platelets, leading to inadequate clot formation. This weakened ability of fibrinogen to interact with platelets may be a critical factor contributing to the observed reduction in clot strength. Further predictive functional analyses indicate that the p.Arg401Gly mutation may impair fibrinogen function by destabilizing the FGG protein. Moreover, electron microscopic observations revealed a sparser fibrinogen structure in patients from this family, validating the compromised capacity of fibrinogen to engage with platelets and thereby prolonging bleeding times. Collectively, these findings underscore a direct relationship between structural abnormalities and functional deficits in fibrinogen, thus providing a significant mechanistic framework for understanding the bleeding tendencies observed in this family.

In conclusion, our study identified a novel mutation in the *FGG* gene (c.1201C > G, p.Arg401Gly) that may contribute to prolonged bleeding times in affected patients. However, since the patients have not undergone any surgical procedures at this point, we cannot conclusively determine whether this mutation might predispose them to thrombotic or bleeding events in the future, particularly during high-risk situations such as surgery. Therefore, closer monitoring and thorough evaluation of patients with such mutations will be essential in clinical practice going forward, to mitigate potential complications.

## Data Availability

The original contributions presented in the study are included in the article/supplementary material, further inquiries can be directed to the corresponding author.

## Footnotes

^1^ https://www.uniprot.org

^2^ https://swissmodel.expasy.org/

^3^ www.geht.org
